# Effects of Secondary Plant Metabolites on Microbial Populations: Changes in Community Structure and Metabolic Activity in Contaminated Environments

**DOI:** 10.3390/ijms17081205

**Published:** 2016-07-29

**Authors:** Lucie Musilova, Jakub Ridl, Marketa Polivkova, Tomas Macek, Ondrej Uhlik

**Affiliations:** 1 Department of Biochemistry and Microbiology, Faculty of Food and Biochemical Technology, University of Chemistry and Technology Prague, Technicka 3, 166 28 Prague, Czech Republic; polivkom@vscht.cz (M.P.); macekt@vscht.cz (T.M.); 2 Department of Genomics and Bioinformatics, Institute of Molecular Genetics of the Czech Academy of Sciences, Videnska 1083, 142 20 Prague, Czech Republic; jakub.ridl@img.cas.cz

**Keywords:** secondary plant metabolites (SPMEs), community structure, carbon flow, bioremediation

## Abstract

Secondary plant metabolites (SPMEs) play an important role in plant survival in the environment and serve to establish ecological relationships between plants and other organisms. Communication between plants and microorganisms via SPMEs contained in root exudates or derived from litter decomposition is an example of this phenomenon. In this review, the general aspects of rhizodeposition together with the significance of terpenes and phenolic compounds are discussed in detail. We focus specifically on the effect of SPMEs on microbial community structure and metabolic activity in environments contaminated by polychlorinated biphenyls (PCBs) and polyaromatic hydrocarbons (PAHs). Furthermore, a section is devoted to a complex effect of plants and/or their metabolites contained in litter on bioremediation of contaminated sites. New insights are introduced from a study evaluating the effects of SPMEs derived during decomposition of grapefruit peel, lemon peel, and pears on bacterial communities and their ability to degrade PCBs in a long-term contaminated soil. The presented review supports the “secondary compound hypothesis” and demonstrates the potential of SPMEs for increasing the effectiveness of bioremediation processes.

## 1. Introduction

Plants as primary producers synthesize tremendous amounts of organic compounds while consuming carbon dioxide and light energy. The spectrum of synthesized compounds is dependent on the plant species, and physiological and environmental conditions. Some of the synthesized compounds are released into the rhizosphere, the soil directly surrounding roots [1,2], which is affected by those released chemicals [3,4,5,6]. Plants deposit approximately 11% of fixed carbon into the rhizosphere [7,8]. The released carbon may appear to represent a significant energy loss for the plant; however, it may actually be beneficial due to the stimulation of biological activity in the rhizosphere [9], including stimulation of rhizosphere bacteria [10], which provide the plant with increased nutrient solubility, fixed nitrogen, and/or competitive suppression of pathogens [11], as well as plant growth promoting molecules [12,13]. Exuded compounds can further change the properties of the surrounding soil and are important for obtaining nutrients, mediating biological interactions, or decreasing the toxicity of pollutants [14,15].

Plant exudates and decomposing litter contain secondary plant metabolites (SPMEs) among other compounds. Beyond their role in mediating plant–microbe interactions, it is hypothesized that SPMEs can stimulate microbial metabolism of pollutants present in the environment, which is termed the “secondary compound hypothesis” [16,17,18]. In a contaminated environment, indigenous microflora usually contain genetic determinants enabling the synthesis of degradative enzymes [19,20]; however, environmental conditions can also often limit natural decontamination processes [21]. Plant-released chemicals can potentially improve these conditions [17,22], induce required genes for degradation of pollutants [23], or serve as primary substrates during the cometabolism of pollutants [16,18]. Although several studies focusing on the effects of SPMEs on microbial diversity and activity towards pollutants have delivered supporting evidence, the introduction of metagenomics [24] and high-throughput sequencing techniques (for review see [25]) has opened up new possibilities for addressing this topic, which has not yet been fully exploited. Despite these advances, linking one specific molecule from exudates with its effect under complex environmental conditions and relationships still remains challenging.

In this review we present the general aspects of rhizodeposition and observed effects of selected secondary metabolites on bacterial cultures. The effects of SPMEs are demonstrated through research that focuses on changes in bacterial community structure and metabolic activity. Further examination of communities in environments polluted by polychlorinated biphenyls (PCBs) and polyaromatic hydrocarbons (PAHs) is presented.

## 2. Root Exudates and Their Effects on Present Microflora

### 2.1. Root Exudates: Carbon Gateway to the Rhizosphere

The rate of all excreted carbon compounds differs along the root in space and time and is dependent on many factors [7]. Initially, photosynthesis was considered the main factor influencing the amount of rhizodeposited carbon, because higher rates of excretion were reported during the day than at night [26]. However, a closer link was later proposed [27], connecting the carbon excretion rate to the rate of transport of the photosynthate into the roots. Therefore, the physiological state of a plant and environmental conditions are considered to be the main drivers of rhizodeposition rates. Another major factor that influences the amount of carbon excreted is the age of the plant: in general, young seedlings release relatively large amounts of carbon, which decreases over time with the increasing age of the plant. For example, rice (*Oryza sativa* L.) seedlings retain about 50% of assimilated carbon in above-ground parts and about 27% is exuded from roots [28]. In rice plants during maturation and flowering, plant carbon management shifts and more carbon is retained in the above-ground parts and less than 4% is exuded [28]. A similar trend was observed for other plants such as wheat (*Triticum* sp.), perennial rye-grass (*Lolium perenne* L.), and mung beans (*Vigna radiate* (L.) R. Wilezek) [29,30,31]. On the other hand, the amount of carbon deposited into the rhizosphere can be increased even in elderly plants under stress conditions as was observed in barley (*Hordeum vulgare* L.): when grown under a low potassium concentration, barley plants considerably increased the amount of exuded organic carbon in slow-growing roots compared to fast-growing roots [32]. The effect of drought, a different type of stress, on crops has been closely studied and an increase of carbon excretion was reported under drought stress in agricultural monocultures [33], although this response is expected to be different for mixtures of species [34]. One potential mechanistic explanation for the increased carbon exudation under stress conditions was proposed to be the loss of integrity of root cell membranes and malfunction of metabolism in affected cells [35]. On average, plants excrete 10%–20% of total assimilated carbon into the rhizosphere over the course of their life span [36,37], with lower amounts of deposition being reported for hydroponic cultures [38] or sterile-grown plants [37].

**Figure 1:**
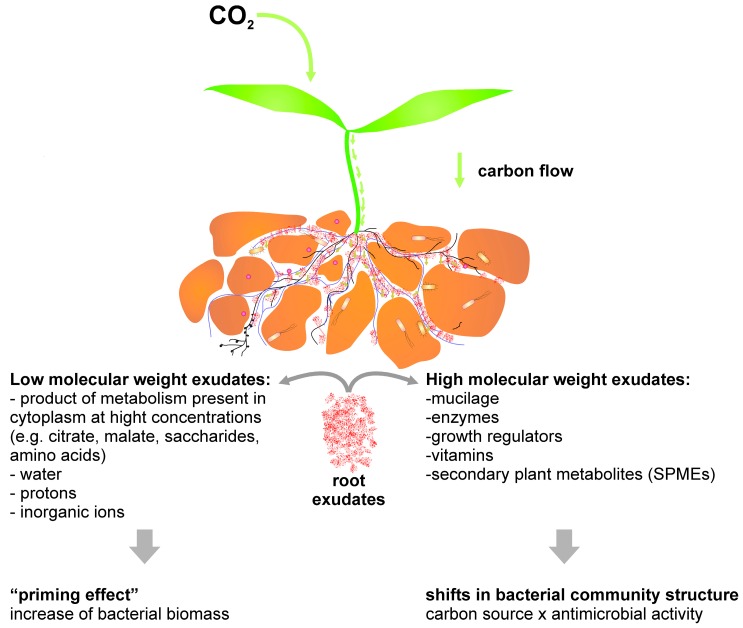
Carbon flow in plants—carbon dioxide is assimilated by plants and used for synthesis of metabolites, which are used in anabolism or released by rhizodeposition into the rhizosphere. Root exudates further affect soil properties and residing microbiota.

**Figure 2:**
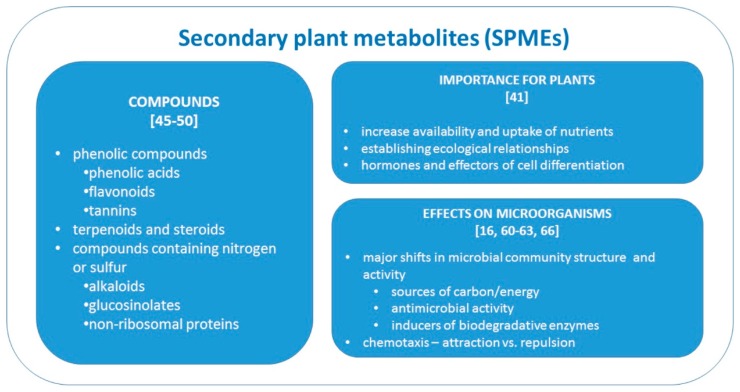
General overview of SPME classification and functions.

Rhizodeposited compounds are products of both primary and secondary metabolism [15] divided into two groups; low molecular weight (L*M*_W_) compounds, which dominate the exudates [27], and high molecular weight (H*M*_W_) compounds (Figure 1). Low molecular weight compounds are composed of water-soluble compounds present in the plant cytoplasm at high concentrations [18]. Other non-carbonaceous L*M*_W_ components include protons, inorganic ions, and water [39]. Because L*M*_W_ compounds are transported via passive transport, they are more prevalent than H*M*_W_ compounds, whose transport requires the input of energy in plant materials. High molecular weight compounds include mucilage, enzymes, growth regulators, vitamins, and many SPMEs (i.e., flavonoids and allied phenolics, terpenoids, and alkaloids) [15,18,40]. Plant secondary metabolites (Figure 2) can have the following mechanistic functions: increasing availability of nutrients or their increased input to the plant, e.g., increase of phosphate solubility or uptake of metals to plants; establishing both positive and negative ecological relationships; acting as hormones or effectors for cell differentiation [41]. General classification of plant chemicals distinguishes primary and secondary metabolites based on whether they have an essential role for metabolism and are ubiquitous in plants [42,43]. However, the given mechanistic functions of SPMEs demonstrate that these metabolites are essential for plant protection from environmental stress, therefore they are important for plants in spite of the wide variety of synthesized compounds among plants [44]. Accordingly, here we consider SPMEs as products distinct from primary metabolism belonging to the following groups based on structure: (i) phenolic compounds including flavonoids; (ii) terpenoids and steroids; and (iii) compounds containing nitrogen or sulfur, such as alkaloids, glucosinolates, and non-ribosomal peptides and proteins [45,46,47,48,49,50]. It should be noted that the role of primary and secondary metabolites can overlap, as is demonstrated in defense against biotic stress, which can be in *Arabidopsis thaliana* mediated by glutathione [51] as well as by various SPMEs derived from indole-like camalexin or glucosinolates [52]. Different organisms do not necessarily use the same molecules for accomplishing the same function such as in metals acquisition, for which the bacterium *Pseudomonas aeruginosa* synthesizes secondary metabolites pyochelin and pyoverdine [53], while some plant roots release citric acid for the same function [54]. Similarly, under high heavy metal content in the environment, plants tend to produce compounds such as phytochelatins [55] or proline [56] to chelate the metals.L*M*_W_ carbon-containing (L*M*_W_-C) components of the root exudates are hypothesized to be the reason for the primary response in rhizosphere microorganisms [14,57], resulting in a “priming effect” on the microbial community—the increase of microbial biomass and soil organic matter decomposition after the input of fresh organic matter [58]. A common example of the priming effect is found in a study that amended multiple soil types with citric acid and found a marked increase in carbon dioxide production and a shift in the relative abundance of β-Proteobacteria [59] in amended soils.

H*M*_W_ SPMEs have also been shown to cause major shifts in soil microbial community structure [60,61,62,63]. Rhizosphere microorganisms respond to SPMEs in different ways; not only can SPMEs serve as carbon and/or energy sources, but they often bear antimicrobial activity or the ability to disrupt bacterial quorum sensing [64,65]—a bacterial system for determination of cell density, which then further influences the gene expression of affected cells. Although some SPMEs have antimicrobial activity and their presence in a system will decrease community richness, some portion of the bacterial community can typically use antimicrobial compounds as a carbon and energy source, as long as the bacteria are not susceptible to the present compounds and those compounds do not reach levels inhibitory to microbial growth [66].

It should be emphasized that not only do plants control the amount of compounds excreted, but also the composition of exudates [67]. For example, if the soil moisture content is very high, oxygen availability will decrease and create anoxic conditions which will then cause plant cells to potentially accumulate lactate and ethanol to phytotoxic levels, and therefore the plant will release larger quantities of these compounds in an effort to decrease their intracellular levels [68]. Exudation patterns of crested wheatgrass (*Agropyron cristatum* (L.) Gaertn. cv. CD-II) were studied under drought, flooding, and nutrient limitation conditions, which revealed that malic acid was predominant among exuded organic acids. Drought led to a significant increase in wheatgrass-exuded organic acids and, under low potassium conditions, an increased amount of exudates was detected. This increase was not caused, however, by exudation of organic acids, suggesting that the wheatgrass released different compounds than acids such as saccharides [69]. During cultivation under phosphate deficiency, bean plants (*Phaseolus vulgaris* L.) increased the amount of exuded phenolic compounds [70], while maize (*Zea mays* L. var. Surprise) increased γ-aminobutyric acid and carbohydrate exudation [71].

Plant metabolites can also enter the rhizosphere through decomposition of deposited litter and below-ground root turnover. Plants annually support the growth of fine roots during spring and summer; however, between 40% and 70% of these fine roots die off in autumn as the plant prepares for winter [72], and these decaying fine roots provide rhizosphere microflora with nutrients and SPMEs. In addition, the decaying roots release air channels in the soil, which allow the increased oxygen flow necessary for most enzymatic activity and can preferentially be used by new roots in subsequent growing seasons [73]. The amount of SPMEs released by root turnover can be substantial, as demonstrated by a study on mulberry plants, which released the same amount of phenolic compounds during root turnover as was exuded by soybeans throughout the growth season [72,74].

### 2.2. Root Exudates: Effect on the Rhizosphere Microflora

It has long been known that there are more microorganisms living in close proximity to plant roots compared to bulk soil; this difference can be up to several orders of magnitude [6,40,75,76,77]. This phenomenon has been termed “the rhizosphere effect” and is potentially due to the increased amount of carbon excreted from roots into the rhizosphere [1,78]. When further examined, differences in microbial densities have been observed in different parts of the rhizosphere and correspond to the amount of exudates released by the particular specialized root cells, which differ along the root. For example, in the wild oat (*Avena fatua* L.) root tips had the highest populations of living rhizosphere bacteria, followed by root hairs; the mature root was the least populated [79].

Changes in exudate patterns not only affect microbial density but also strongly affect the structure [80,81] and function of microbial communities. These shifts then alter plant metabolic pathways and regulations [82,83], which then can retroactively result in changes of exuded compounds [84]. These mutually affected relationships across microbial communities and plants arise from the need for competitive regulation, because both the plant and soil microorganisms depend on resources present in the same soil.

Root exudates can play an important role during chemotaxis by serving as attractants or repellents for soil microbiota [75]. For example, a strain of *Rhodococcus erythropolis* has been found to be attracted to phenolic compounds exuded from *Arabidopsis thaliana* L. roots [85], and phenolic acid root exudates have been shown to play a very important role in the nodulation process by serving as attractants for *Rhizobium* species [86,87]. Chemotaxis of beneficial microflora is also important for disease suppression in some plants due to competitive colonization of plants by symbionts protecting the host plant from pathogenic bacterium. One example of this is the plant symbiont *Pseudomonas fluorescens*, which is attracted to citric and malic acid released by tomato (*Lycopersicon esculentum* L.) roots [88] and can protect the host plant from a pathogenic bacterium, *Ralstonia solanacearum*, which is attracted to its host by diverse amino and organic acids and different SPMEs [89]. However, the same combination of SPMEs, amino acids, and other exuded compounds that attracts the beneficial bacteria also serves to attract potentially pathogenic organisms [90]. In addition to SPMEs and attracted microorganisms, plant resistance to a disease can also be influenced abiotically by available nutrients such as essential ions or nitrogen [57].

### 2.3. Roots and Associated Microorganisms: How to Study Interactions?

As mentioned above, the rhizosphere is shaped by complex interactions between plants and microorganisms, and the study of these interactions requires interlacing many different experimental approaches [91] (for an overview and examples, see Table 1).

Growing roots are the main drivers of changes in the rhizosphere and therefore methods for studying their growth and morphology in situ have been developed such as root windows installed along the soil profile beneath studied plants [92], with imaging systems collecting data over a certain time period [93]. However, this approach visualizes root development on the observed interface and does not provide information on the whole root system of the studied plant. In order to access the 3D structure of a root system, transparent culture media suitable for plant cultivation have been developed [94], examples of which include Phytagel™ and Nafion™ [95]. Although useful for visualizing root growth, these artificial and clear culture media have different composition and physical properties than soil, which can have artificial influences on root growth. For example, roots grow faster and thinner when the penetration resistance is low [96]. In order to overcome such limitations in soil, computed tomography (CT) [97,98], magnetic resonance (MRI) [99,100], and neutron radiography [101] have been successfully implemented in order to provide images of root system with high resolution (for an extensive review of imaging technologies see Downie et al. [102], Oburger et al. [94], and York et al. [6]).

Implementation of high-resolution imaging techniques has also allowed for tracing nutrient transport in plants following their release into the rhizosphere. For example, MRI [103] and neutron tomography [102] have been successfully used to monitor water uptake by roots. Chemicals in the rhizosphere have been traced by ^11^C positron emission tomography (^11^C-PET) combined with MRI, which allowed for tracing newly synthesized photosynthate throughout the plant and rhizosphere [104]. Tracing of chemical compounds in the rhizosphere is also possible using optodes—selective optical sensors—which have been implemented in studying oxygen gradient, pH, and CO_2_ dynamics in different plants [105,106,107,108].

Tracing of exuded compounds, including their uptake by rhizosphere microflora, can be studied through fluorescence-based methods. One fluorescence-based method uses biosensors—genetically modified (GM) microorganisms that express fluorescent proteins or are able to emit bioluminescence in the presence or absence of a studied molecule. The main advantage of such sensors is specificity in detection of studied microorganisms and compounds; however, GM microorganisms are distinct from the wild type and therefore may behave in different ways [109] (for a review on biosensors and their environmental application see Jusoh et al. [110]). In the past, biosensors have been developed for studying carbon flow in plant exudates [111,112], during nodulation [113], and for tracing bacterial quorum sensing [114] and root colonization [115]. In addition, biosensors have been used for tracing chemical compounds like nitrate [116], nitrogen [117], phosphorus [118], arsenic [119], saccharides and amino acids [120], and iron [121]. The light signal produced by a biosensor can also be detected in situ using fluorescent microscopy. Fluorescence in situ hybridization (FISH) is a microscopy method used for quantification of populations bearing selected genes [122,123]. This method is especially useful for studying biocontrol of plant pathogens [124,125,126].

Isotope probing methods based on radioisotopes (radioisotope probing, RIP) or stable isotopes (stable isotope probing, SIP) have been shown useful for determination of rhizodeposition rates and carbon flow from the plant into the rhizosphere. These techniques require incubation of plants in atmosphere-containing ^14^CO_2_ [22] or ^13^CO_2_ [127], respectively, and exudates or microorganisms feeding on the now labeled exudates can be determined. These microorganisms capable of utilizing labeled substrates can then be detected based on separation of biologically important markers with an incorporated “heavy” or radioactive isotope and further analysis of those labeled biomarkers [128,129,130,131]. Examples of biological markers that can be labeled in such experiments are phospholipid-derived fatty acids (PLFA), DNA, RNA, or proteins that can be analyzed by various methods. For direct observation of microorganisms interacting with radiolabeled plant exudates, FISH combined with microradiophotography (FISH-MAR) can be used [122]. For analysis of the plant-associated microbial community structure based on DNA or RNA, metagenomics [132] or metatranscriptomics [133], respectively, can be exploited. Metagenomics studies the complete DNA material present in an environment [134] and is commonly combined with high-throughput sequencing, while metatranscriptomics focuses solely on all present RNA molecules and thus is more suitable for evaluation of microbial response to environmental stimuli [135]. In other words, metagenomics can answer questions about which microorganisms reside in the rhizosphere and what is their metabolic potential (i.e., which functional genes the microorganisms have), while metatranscriptomics answers the question of which genes are expressed under studied conditions. In addition, metagenomics and metatranscriptomics can be dived into two groups: (i) unselected metagenomics and metatranscriptomics, which both focus on random sequencing of all informational molecules in the samples; and (ii) targeted metagenomics and metatranscriptomics, which both focus on a reduced pool of informational molecules and can be targeted based on sequence (in this form, high-throughput sequencing is called amplicon sequencing) or function [136]. In addition to metagenomics and metatranscriptomics, two other methods for studying plant–microbe interactions have been developed namely: (i) metaproteomics studying all proteins in an environmental sample (for review see Hettich et al. [137]); and (ii) metabolomics dealing with small molecular weight molecules present during cell metabolic processes (e.g., saccharides, amino acids, fatty acids, vitamins, secondary metabolites (for review see van Dam et al., [138])).

## 3. Role of Secondary Metabolites in Biodegradation of Organic Contaminants

### 3.1. Secondary Metabolites: Structural Similarities to Organic Pollutants

**Figure 3:**
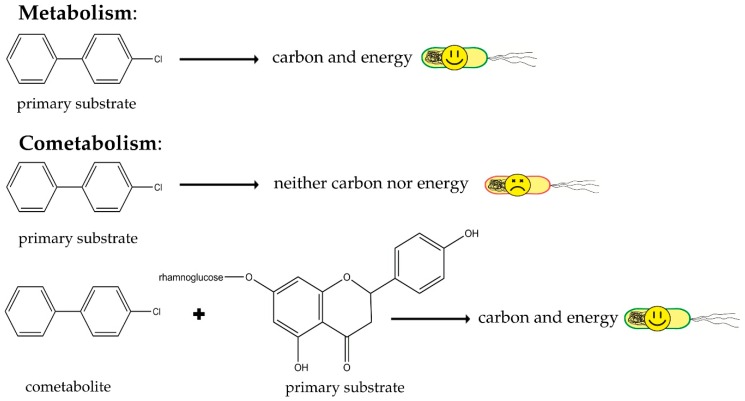
Schematic visualization of cometabolism, differences between primary substrates and cometabolites, and role of secondary plant metabolites (SPMEs) in cometabolism of pollutants.

**Figure 4:**
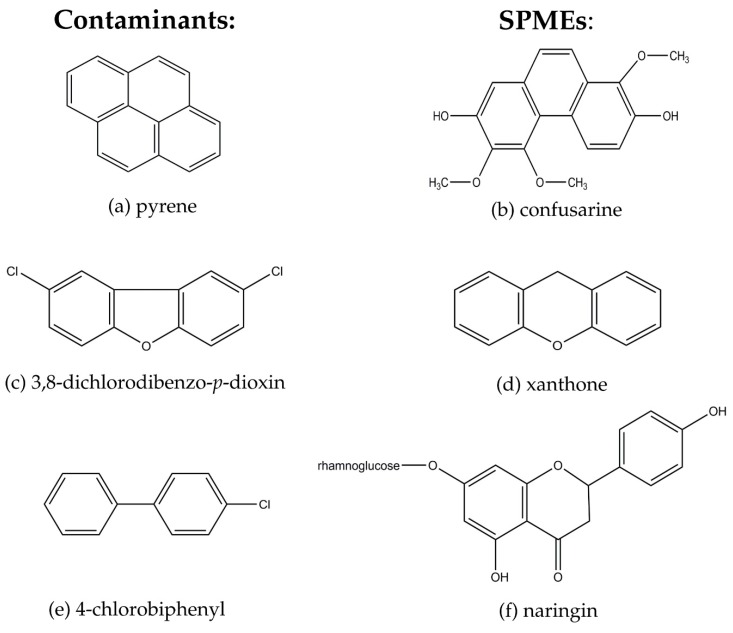
A few examples of structural similarities between contaminants and plant secondary metabolites (SPMEs), adapted from Singer et al. [18]. (**a**) pyrene; (**b**) confusarine; (**c**) 3,8-dichlorodibenzo-*p*-dioxin; (**d**) xanthone; (**e**) 4-chlorobiphenyl; (**f**) naringin.

As soon as a contaminant enters the environment, the microbial community reacts and begins to adapt to its presence. The organisms able to use the contaminant as a carbon/energy source or terminal electron acceptor will prosper and become more dominant in the community [157]. In order to metabolize the present contaminant, microorganisms must synthesize specific enzymes, the production of which usually requires an inductor such as the contaminant [158,159,160,161]. Some contaminants cannot serve as the primary substrate and provide cells with energy, and therefore are degraded via cometabolism with another compound, which does serve as the primary substrate (Figure 3) [162,163]. Some plant-derived compounds present in the rhizosphere can serve as primary substrates in cometabolism or inducers of degradative enzymes due to their structural similarities to the contaminants (Figure 4) [18,164], and therefore may provide the energy needed for contaminant metabolism [16,165].

The presence of large amounts of SPMEs in the rhizosphere, possibly serving as primary substrates and/or enzyme inducers, can explain the increased amount of microorganisms capable of degradation of pollutants that can be found in the rhizosphere versus the bulk soil [18,166]. Yet the spectrum of exuded compounds differs among plants and therefore stimulation of degradation may not be equal for all compounds and microorganisms [72]. Donnelly et al. [16] were the first group to demonstrate the ability of SPMEs to support the growth of contaminant-degrading bacteria and to enhance the bacterial degradative activity towards polychlorinated biphenyls (PCBs). After their work, many additional experiments demonstrating the stimulation of degrading activity towards PCBs have been published [63,167,168,169]. The effect of plants or their metabolites is usually compared to the most efficient inductors known and applied in laboratory experiments, such as biphenyl, which is a commonly used inductor of the PCB-degradation pathway [170,171]. In addition to PCBs, other pollutants have been identified that are more efficiently transformed in the rhizosphere than in the bulk soil, including PAHs and other petroleum hydrocarbons (PHs), pesticides, detergents, and explosives [172].

### 3.2. Phenolics: From Simple Phenolics to Flavonoids and Lignin, Their Biological Role and Effect on Biodegradation of Pollutants

Phenolic compounds represent a very diverse group of SPMEs. Their biosynthesis proceeds in a manner similar to the production of aromatic amino acids via the shikimate pathway, which occurs only in plants and microorganisms [173]. The main metabolite in this pathway is shikimic acid, which serves as the precursor of amino acids as well as benzoic and cinnamic acid [174]. Further modifications of these acids lead to lignins, lignanes, phenylpropenes, and coumarines [174]. Derivatives of cinnamic acid can be reduced, which leads to the synthesis of phenylpropanoids, or hydroxylated and used for the synthesis of coumarines or psoralens [175]. Using the polyketide biosynthetic pathway, cinnamic acid is transformed into 4-coumaric acid, which condensates first with acetyl coenzyme A and then with three combined acetate units derived from malonyl-CoA, leading to the synthesis of stilbens, which can be further extended to flavonoids. Flavonoids are widespread and very often occur in the form of glycosides. Most flavonoids compose of phenylbenzopyrone core and differ in the number and position of hydroxyl groups [176].

When cinnamic acid is derivatized, caffeic, ferulic, or sinapinic acid is produced and can lead to the biosynthesis of lignin, among other compounds. The chemical composition of monomers used is specific to the plant species, but usually alcohols derived from cinnamic acid (e.g., coniferyl and sinapyl alcohol) are polymerized by peroxidases, which catalyze hydrogenation of the alcohols followed by creation of free radicals freely joining together and creating lignin. Lignin and lignocellulose are some of the most widespread components of plant biomass [177] and their microbial degradation plays an important role in the carbon cycle [178]. Lignocellulose is composed of cellulose, hemicellulose, and lignin, which are linked by both covalent bonds and non-covalent interactions. These non-covalent interactions lead to the creation of a complex three-dimensional structure, which is resistant to chemical and biological degradation [179]. Nevertheless, metabolic pathways involved in lignin depolymerization have evolved in microorganisms. Although some bacteria are involved in lignin biodegradation [180,181], degradation by ligninolytic white rot fungi has been investigated in closer detail [182].

The functional group of white rot fungi belong mostly to the Basidiomycetes class. The name “white rot” is derived from the white color, which appears on wood after the fungi degrade lignin while leaving cellulose behind. Microbial degradation of lignin proceeds extracellularly and is mediated by enzymes with low substrate specificity, such as peroxidases and laccases. The peroxidases participating in lignin degradation are expressed under nitrogen-limiting conditions [183,184] and are divided into three groups: lignin peroxidases (LiPs, EC 1.11.1.14), manganese peroxidases (MnPs, EC 1.11.1.13), and versatile peroxidases (VPs, EC 1.11.1.16). LiPs are typically present in the form of isoenzymes which are able to depolymerize lignin via oxidation of veratryl alcohol and creation of its radical cations, which then leads to oxidation of not only phenolic compounds but also a wide variety of compounds including even non-phenolic compounds like amines, aromatic ethers, or polycyclic aromatic compounds. MnPs are structurally similar to LiPs [185]; however, they depolymerize lignin by a slightly different mechanism, namely via the release of Mg^3+^-oxalacetate, which selectively oxidizes phenolic compounds and creates phenoxy radicals indirectly responsible for lignin oxidation. VPs have been reported to dispose of both the activities of LiPs and MnPs. Other examples of enzymes that can have a role in lignin degradation are cytochrome P-450 monooxygenases [186], enzymes oxidizing cellobiose, dehydrogenating amyl alcohol derivatives, or quinone reductases [177,187]. In addition to peroxidases, other proposed indirect mechanisms of lignin degradation are based on Fenton’s reaction (i.e., the creation of hydrogen peroxide with Fe(II) ions) and yield hydroxyl radicals. The hydroxyl radical is a strong non-selective oxidant and is involved in lignin modification and polysaccharide degradation [188]. The hydrogen peroxide necessary for the proposed mechanism has been shown to be produced by several white and brown rot fungi [177], either by the production of small phenolic compounds reducing iron and leading to H_2_O_2_ [188] or by enzymatic activity, e.g., aryl-alcohol oxidase (EC 1.1.3.7) or cellobiose dehydrogenase (EC 1.1.99.18) [177].

In some white rot fungi, ligninolytic culture conditions have often been associated with the degradation of PCBs of PAHs (for an overview see Table 2). Many studies reported the degradation of PCBs by *Pleurotus ostreatus* under both nitrogen-limiting and -rich conditions [189], and by *Phanerochaete chrysosporium* only under nitrogen-limiting conditions [190]. However, the degradation of PCBs in *Phanerochaete chrysosporium* was proved to be mediated by radical attack and not due to enzymatic activity [191]. Other examples of the contaminant-degrading capabilities associated with white rot fungi include reported degradation of certain PAHs by liquid culture of *Irpex lacteus* [192,193] or by *Pleurotus ostreatus* [194], which also stimulated the growth of Actinobacteria capable of the degradation of PAHs [195]. Later, MnPs were also reported to be involved in the degradation of PAHs [196]. In addition, the fungus *Lentinus tigrinus* [197] was described as capable to degrade PAHs using laccase under nitrogen-rich conditions and MnP under nitrogen-limiting conditions. The analysis of degradation products of PAHs indicated activity of cytochrome P-450 in the early stages of cultivation, suggesting that it might be used in the initial hydroxylation of those substrates in fungi. White rot fungi were also reported to be able to degrade other organic pollutants like polychlorinated dibenzo-*p*-dioxins [198], trinitrotoluene [199], lindane [200], and pentachlorophenol [201].

Some bacteria, such as actinomycetes, also possess the ability to degrade lignin [214]—although, unlike some fungi, they do not carry genes for complete depolymerization and biotransformation of lignin. In addition, the bacterial peroxidases are reported as less powerful for lignin oxidation [181]. One of the enzymes possibly used for bacterial extracellular oxidation is a dye-decolorizing heme peroxidase that is able to oxidize non-phenolic components of lignin. This peroxidase, among other degradative enzymes, has been found in *Rhodococcus jostii* RHA1 [215], a well-described potent degrader of organic contaminants [216].

In addition to water conduction and supporting structure, lignin is used for harmless storage of partially metabolized contaminants in plants. Ligninolytic activity of microorganisms leads to repeated release of lignin-building units including phenolic compounds as well as partially plant-metabolized contaminants, which are usually more polar and bioavailable [217] and can be further transformed. For instance, chlorobiphenyls have been shown to be hydroxylated in plant cells [218] and these hydroxy-derivatives have been shown to be further transformed by enzymes of the biphenyl degradation pathway [219,220,221,222], supporting the hypothesis that biphenyl-degrading bacteria play a role in the degradation of final products derived from lignin [171]. More recently, evidence was provided that the biphenyl catabolic pathway evolved in some bacteria to allow for the metabolism of SPMEs in soil [223].

The connection between phenolic compounds and the biphenyl catabolic pathway has been the focus of studies for decades (for an overview see Table 2). Donnelly et al. [16] tested the growth of three biphenyl-degrading bacteria, *Cupriavidus necator* H850 (formerly *Alcaligenes eutrophus*), *Burkholderia xenovorans* LB400 (formerly *Pseudomonas* sp.), and *Rhodococcus globerulus* P6 (formerly *Corynebacterium* sp. MB1), on 13 phenolic SPMEs serving as a sole carbon source. For *Cupriavidus necator* H850, several compounds including apigenin, catechin, or morin served as a better growth substrate than biphenyl, and the best cometabolic degradation of PCBs was observed while growing on naringin. For the strains LB400 and P6, the fastest growth was found during cultivation with biphenyl as the sole C-source; however, these strains were also able to utilize some phenolic compounds like myricetin, catechin, or chrysin as growth substrates. The highest PCB-degradation activity was observed for *Burkholderia xenovorans* LB400 during growth on myricetin as the sole C-source and for *Rhodococcus globerulus* P6 on coumarin. In addition, the strain *Cupriavidus necator* H850 has been shown to use salicylic acid as a growth substrate and inducer of PCB cometabolism [167]. In a more recent experiment, Toussaint et al. [85] studied the ability of root exudates from *Arabidopsis thaliana* to support the growth of *Rhodococcus erythropolis* U23A and induce PCB-degradation activity. Although the bacterium was able to utilize concentrated root exudates as a sole growth substrate, single flavonoids detected as main components in the exudates were not able to support the growth of the bacterium. Nevertheless, when *Rhodococcus erythropolis* U23A was grown on root exudates as a sole carbon source, it was able to cometabolically convert 4-chlorobiphenyl to 4-chlorobenzoic acid and metabolize three of 18 tested PCB-congeners. Flavanone was detected as the most abundant compound in the root exudates and its ability to induce the biphenyl degradation pathway in U23A was confirmed. This research demonstrated the ability of *Arabidopsis thaliana* root exudates to increase the level of biphenyl-catabolizing enzymes above the basal level. In a follow-up experiment [224], growth and induction conditions were optimized, and flavanone, flavone, and isoflavone were detected as significantly better inducers of biphenyl pathway than biphenyl itself. However, the primary growth substrate increased the efficiency of the expression of biphenyl degradation pathway, as can be demonstrated in the case of isoflavone, which was a strong inducer during the growth on sodium acetate, mannitol, and sucrose, but its induction capability was much weaker during the growth on glucose or mannose [223].

The ability of plant-derived phenolics to increase degradation of pollutants beyond PCBs has also been the subject of research [225,226,227,228,229] and can be demonstrated by an experiment by Scheublin et al. [211], who investigated the transcription profiles of a Gram-positive bacterium *Arthrobacter chlorophenolicus* A6 after the growth on leaves of common bean (*Phaseolus vulgaris*) and on tryptic soy agar (TSA) with and without the addition of 4-chlorophenol (4-CP). The authors discovered that a subset of *cph* genes for the degradation of 4-CP was expressed after the growth on the leaves. It was hypothesized that the genes encoding for enzymes transforming hydroquinone to 3-oxoadipate were induced by hydroquinone detected in leaf washes.

### 3.3. Terpenes: Biological Role and Effect on Biodegradation of Pollutants

Terpenes are a class of natural compounds, mostly of plant origin, which are composed of two or more isoprene units. The biological synthesis of terpenes begins with the condensation of isopentenyl diphosphate and dimethylallyl diphosphate, which are both synthesized from three acetyl coenzyme A units at the beginning of the mevalonate pathway. Terpenes are classified based on the number of condensed isoprene units and are responsible for characteristic plant fragrances and essential oils in many fruits (e.g., citrus fruits) and herbs; they can also act as chemoattractants or repellents [230].

Generally, microbial uptake of terpenes is challenging due to their volatility, low water solubility, and common antimicrobial properties. Nevertheless, at the beginning of the 1960s a bacterium *Pseudomonas* sp. capable of growth using d-limonene, one of the most common terpenes, as a sole carbon source was described [231]. After this discovery, other bacteria capable of terpene utilization have been described [232,233,234,235]. To date, five different microbial biotransformation pathways for limonene have been proposed [236]. The main utilization pathway starts with the hydroxylation of C7 and yields in perillic alcohol, which is further oxidized to perillic acid and further metabolized via pathway similar to β-oxidation. The ability to metabolize d-limonene through perillic alcohol does not seem to be species-specific as it was described in micromycetes (*Hormonema* sp. [237]), yeasts (*Yarrowia lipolytica* [238]), and bacteria (*Bacillus stearothermophilus* BR388 [239] or *Pseudomonas* spp. [231,240]). One of the other biotransformation pathways results in the production of (3*R*)-3-isoprenyl-6-oxoheptanoyl-CoA, as was described in bacterium *Rhodococcus erythropolis* DCL14 [236]. This bacterium, similarly to *Pseudomonas fluorescens* [241], can metabolize limonene through limonene 1,2-epoxide and limonene 1,2-diol. Another hydroxylation leads to hydroxyl ketone, which undergoes transformation via a Baeyer–Villiger reaction mediated by an oxygenase. The resulting product is then metabolized through β-oxidation [236]. Other widespread terpenes are α- and β-pinene, which can be found in pine bark and needles, and represent byproducts formed during cellulose synthesis. The bacterium *Pseudomonas* sp. PIN has been implicated in the metabolism of pinene via limonene, although the exact pathway for this metabolism is unclear [232]. However, a large amount of perillic acid was detected, suggesting that *Pseudomonas* sp. PIN uses the same metabolic pathway for the transformation of limonene as *Rhodococcus erythropolis* DCL14.

From the wide group of terpenes, limonene, carvone, and pinene have been the most intensely studied as possible primary substrates for cometabolic degradation of pollutants (for an overview see Table 2). Tandlich et al. [168] investigated the possible induction effect of limonene and carvone in bacterium *Pseudomonas stutzeri*, a known degrader of PCBs. The strain was grown on xylose or glycerol used as carbon sources; the terpenes were used in concentrations of 10 and 20 mg·L^−1^ and the degradation of formerly used PCB-mixture Delor 103 was analyzed. The results showed that, after induction, the bacterium degraded a higher amount of PCBs during the growth on xylose in comparison to the growth on glycerol, with the most promising system being cultivated on xylose and induced by carvone. Though PCB degradation during growth on glycerol was lower, a broader spectrum of congeners was degraded. When the bacterium *Pseudomonas stutzeri* was induced by limonene, it degraded an increased amount of higher-chlorinated congeners and the increase in degradation was dependent on the concentration of the inducer.

In contrast to the Tandlich study, Gilbert et al. [207,208] found that carvone derived from mint (*Mentha spicata*) induced PCB degradation by *Arthrobacter* sp. B1B. Carvone in combination with surfactants has also been investigated in an effort to increase the bioavailability of Aroclor 1242—a commercial mixture of PCBs—for microbial degradation by bacterial strains *Arthrobacter* sp. B1B and *Cupriavidus necator* H850 in artificially contaminated soil [167]. Application of carvone-grown *Arthrobacter* sp. B1B in combination with (i) sorbitan trioleate in ratio 1:10; (ii) *Cupriavidus necator* H850 in ratio 1:1; or (iii) fructose in a 1:10 ratio led to a doubled degradation of PCBs in soil in comparison with non-bioamended controls after 18 weeks of repeated amendment. The differences in removal of PCBs were not significant among different amendments; however, sorbitan trioleate was demonstrated to support growth of the inoculum and increase the bioavailability of PCBs and degradation of higher-chlorinated congeners. Co-inoculation of both strains did not lead to enhanced removal of PCBs; nevertheless, an increase in degradation of several multiple *ortho*-substituted congeners was detected.

Terpenes have also been demonstrated to induce cometabolism of pollutants other than PCBs. For example, degradation of 2,4-dichlorophenol (2,4-DCP)—product of 2,4-dichlorophenoxyacetic acid metabolization—was studied after the addition of limonene and α-pinene in three different soil types [210], one of which was sampled from grassland covered bog, and the others from below pine and oak trees. Mineralization of 2,4-DCP was comparable among samples, but α-pinene showed a better effect than limonene in soil derived from pine surroundings and bog, suggesting that the bacteria in those environments may be better adapted to α-pinene’s presence. As another example of chlorophenolics, pentachlorophenol (PCP) has been proposed to be metabolized by *Arthrobacter* sp. B1B induced by l-carvone [242] in the same manner as PCB mixtures [208]. Furthermore, cumene (isopropylbenzene) was demonstrated as a suitable growth substrate for enrichment cultures of bacteria capable of degradation of trichloroethylene (TCE) [213]—a widespread water contaminant originating from extensive use of chlorinated solvents. Cumene induced TCE degradation capability in the bacterium *Rhodococcus gordoniae* P3, which was detected both in pure liquid culture and soil [212]. Later, cometabolic biotransformation of TCE was demonstrated using other terpenes and terpenoids, namely *R*-carvone, *S*-carvone, linalool, and cumene as growth substrates for indigenous bacterial communities from a site contaminated by TCE in the UK [243].

## 4. Complex Effect of Plant Metabolites on Bioremediation of Contaminated Soil

The secondary compound hypothesis suggests that SPMEs released into the environment by root exudation or plant litter decomposition affect soil microbial populations and can stimulate their metabolic activities toward degradation of organic contaminants [16,17,18]. Although we have presented evidence supporting this hypothesis, these studies were based mostly on experiments with pure cultures. With the implementation of high-throughput sequencing technologies, researchers are able to assess the whole community structure in any environment; therefore, the method is suitable for the assessment of the role of SPMEs in the entire microbial community [244,245]. Through the development of SIP, researchers have gained the ability to directly link microbial community structure and function based on incorporation of ^13^C, ^15^N, or ^17^O derived from labeled compounds [128,129,246]. These microbial ecology techniques have contributed to a successful understanding of ongoing processes in contaminated sites, which is important for the improvement of bioremediation processes (for an overview of this section see Table 2).

The effect of individual SPMEs on microbial community structure and degradation of PCBs in a long-term contaminated soil [245] was investigated in response to eight-week repeated soil amendment with limonene, naringin, and caffeic acid [63,247]. Bacterial diversity was reduced in all samples compared to the control/non-amended soil, with caffeic acid being associated with the largest reduction in community diversity. The metabolism of 4-chlorobiphenyl in the bacterial community revealed activity of Proteobacteria in all samples; however, differences at lower taxonomic levels were detected. In non-amended soil, active populations of *Pseudomonas*, *Rhodanobacter*, *Azoarcus*, *Porphyrobacter*, and *Gemmatimonas* were detected, from which only *Azoarcus* was detected in amended soils, namely in soil amended with limonene. Otherwise, the soils with limonene and naringin additions were dominated by *Hydrogenophaga* genus, while the soil amended with caffeic acid was found to be dominated by the genus *Burkholderia*. Additionally, patterns of degraded PCB congeners among the samples differed and, although caffeic acid-amended soil harbored less diverse bacteria, a broader spectrum of degraded congeners was detected. Importantly, degradation of usually very persistent higher-chlorinated biphenyls was detected.

**Figure A1:**
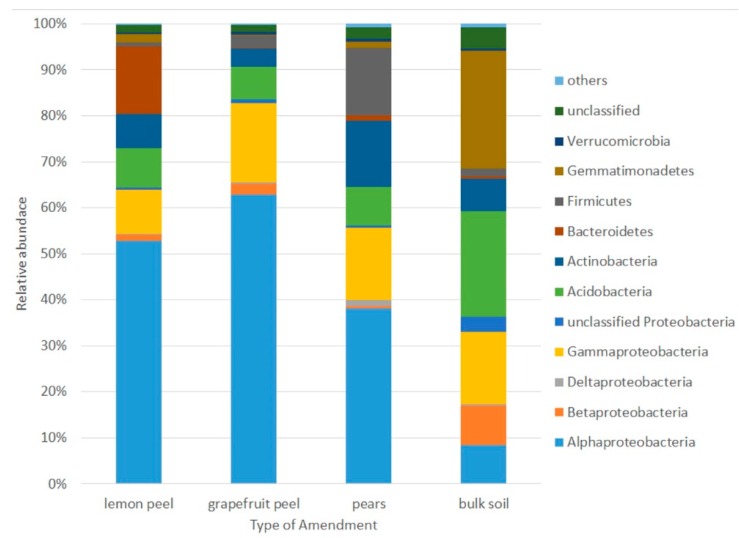
Total community composition in soils after amendment with natural materials represented as bacterial phyla or classes in the case of Proteobacteria. Bulk soil was not amended by any material.

SPMEs are commonly present in soil as a complex mixture, with different SPMEs having differential impacts on the microbial community. One study examined the effects of a complex mixture of SPMEs from plant litter on the biodegradation of Aroclor 1242 in soil. Hernandez et al. [61] amended soil with either orange peel, ivy or eucalyptus leaves, or pine needles and assessed the disappearance of PCBs over six months. Under these amendment conditions, complete mineralization of present PCBs was achieved, while degradation of only lower chlorinated congeners was detected in the control sample. Additionally, all types of soil amendment led to a five-fold increased abundance of cultivable biphenyl-utilizing bacteria [16]. Following the study by Hernandez et al. [61], it is relevant to present new insights from a study focused on the effects of grapefruit peel, lemon peel, and pears on the changes in bacterial communities and their PCB-degradation activity in long-term contaminated soil (for a more detailed description of the methods used, see Appendix A). The plant litter used was rich in naringin, limonene, and caffeic acid, respectively [248]. Additionally, these SPMEs have already been associated with an enhancing of degradative activity towards PCBs [63]. Our results confirmed that the natural materials rich in selected SPMEs changed the community structure, as can be seen in Figure A1. This observation was further supported by obtaining phylogenetically different and more abundant biphenyl-utilizing isolates during cultivation, which is a commonly reported phenomenon [61,203]. Although the amount of cultivable biphenyl-utilizing bacteria increased, the diversity in soil decreased in the following order: the untreated soil was the most diverse, followed by soil samples treated by pears and grapefruit peel, with the soil amended with lemon peel being the least diverse. A decrease in species richness can in some cases lead to the inability of an ecosystem to keep providing its services (e.g., biomass turnover) [249] and was reported as significantly influenced by plant litter [250]. However, in this experiment, the ability of present microflora to degrade 4-chlorobiphenyl and benzoic acid was retained, as was demonstrated by SIP. Different bacteria deriving carbon from 4-chlorobiphenyl and benzoic acid were detected in each treatment. The incubation of soil with natural materials led to changes in the patterns of detected PCB congeners, suggesting an ongoing, preferential biodegradation process [247]. These results demonstrate a possible usage of lemon and grapefruit peels, which are typically a waste product from juice production, for bioremediation of contaminated sites. Both presented experiments demonstrate that SPMEs derived from plant litter affect soil microflora similarly as plant-exuded SPMEs with the possibility to stimulate degradative activities towards PCBs.

As was described earlier, the root turnover is an important source of SPMEs in soil. The effect of 43 different plants’ root tissues on the removal of the PAHs pyrene and benzo[*a*]pyrene from soil was tested by Yi and Crowley in a series of experiments [209]. From all the tested plants, only four stimulated degradation, namely radish (*Raphanus sativus* L.), potato (*Solanum tuberosum* L.), carrot (*Daucus carota* L.), and celery (*Apium graveolens* L.). In these plants, the authors further focused on the compounds probably responsible for the stimulation of degradation activity in the soil. In celery, the most effective stimulator in the experiment, terpenes and derivatives of salicylic acid were identified as the specific compounds responsible for the increase in removal of PAHs, though the application of these SPMES in their pure form did not prove successful for degradation of PAHs, indicating that an unspecified and uninvestigated synergistic effect was causing the degradation activity. Therefore, a more generic approach was considered and linoleic acid (an unsaturated C18:2 fatty acid) was detected as the only common factor in the measured plants. After these findings, the authors conducted an experiment focused on the comparison of rhizodeposition by celery (*Apium graveolens* L.) and wheat (*Triticum aestivum* L.), celery root crushate, and the addition of linoleic acid or its sodium salt. In the pots with celery plants or root crushate, and in those with linoleic acid or sodium linoleate, the degradation of benzo[*a*]pyrene and pyrene was fast and comparable among the treatments. On the other hand, wheat did not enhance the removal of PAHs as their residual amounts were comparable to the unplanted control. Although the role of linoleic acid in the stimulation of degradation of PAHs was not the topic of the presented research, the authors suggested it might increase the number of degrading microorganisms, or more likely serve as a biosurfactant increasing the bioavailability of PAHs.

Uhlik et al. [202] have also investigated the potentially stimulatory effects of growing certain plants in contaminated soils, namely the effect of horseradish (*Armoracia rusticana* P. Gaertn., B. Mey. et Scherb.) grown in soil contaminated with PCBs on bacterial communities in the rhizosphere deriving carbon from biphenyl. Changes in composition of bacterial communities after planting horseradish into the soil were detected; in the rhizosphere, only proteobacterial sequences were detected to derive carbon from biphenyl, while the bulk soil contained in addition sequences mostly belonging to Firmicutes. Looking for a suitable plant capable of stimulating microbial PCB degradation during remediation process, Leigh et al. [203] focused on autochthonous PCB-degrading bacteria associated with mature trees naturally colonizing a PCB-contaminated site and changes in their abundance in dependence to seasonal changes and soil depth. No significant differences were detected among the samples in the uppermost layer, which contained roots of widespread grasses and forbs in addition to the tree (Austrian pine—*Pinus nigra* J. F. Arnold, ash—*Fraxinus excelsior* L., two weeping birches—*Betula pendula* Roth, goat willow—*Salix caprea* L., or black locust—*Robinia pseudoacacia* L.) roots. In the middle soil layer, where only tree roots were present, again no significant differences were detected between June and August. However, in samples collected in November and May, a significantly higher number of PCB-degraders was isolated from the pine root zone. The deepest layer of willow root zone harbored the highest amount of PCB-degraders. These results suggest that Austrian pine and willow support the growth of PCB-metabolizing bacteria and can be suitable candidates for rhizoremediation. Therefore, Leigh et al. [251] studied the pine root zone microbiome in closer detail, focusing on bacteria deriving carbon from biphenyl and their functional genes. When the ^13^C-labeled metagenome was analyzed using GeoChip functional array [252], 28 different genes associated with the degradation of aromatic hydrocarbons were detected, revealing several genes of the β-ketoadipate pathway, which is common in soil bacteria during microbial degradation of many SPMEs.

As was mentioned above, the amount and composition of root exudates is dependent on environmental conditions [26,33]. Therefore, some trees growing in higher latitudes have been reported as producing a higher amount of secondary metabolites than similar tree species growing at lower latitudes [253], which could possibly lead to increased stimulation of degradation capabilities of present microflora. In 1996, a bioremediation project was initiated in Alaska with soil contaminated by diesel and crude oil, vegetated with annual ryegrass (*Lolium multiflorum* Lam.) alone or in a mixture with red fescue (*Festuca rubra* L.), and fertilized. After two years, plots that had been vegetated and fertilized had significantly increased petroleum hydrocarbon loss when compared to unamend sites [205]. Fifteen years later, Leewis et al. [254] reexamined the site and described the long-term effects of phytoremediation and nutrient amendment on the site. In a preliminary screening, a decrease of 80%–95% in the last reported values of contaminants was detected, which suggested ongoing bioremediation processes at the site. In addition, with an increase in trees, a decrease in petroleum hydrocarbons concentration was observed, and the diesel contaminated site was colonized by a higher amount of plants. Interestingly, the non-native annual grasses were not found on the site anymore; only native trees and seedlings were detected with willow (*Salix* sp.), Alaskan birch (*Betula neolaskana*), white spruce (*Picea glauca* (Moench) Voss), and balsam poplar (*Populus balsamifera* L.) dominating the site. When they assessed microbial diversity, the researchers reported patterns dependent on the original applied soil treatments.

## 5. Conclusions and Future Perspectives

Secondary metabolites present in plant fruits, leaves, or exudates alter the composition of bacterial communities and their metabolic pathways. These interactions can be potentially exploited to increase the effectiveness of bioremediation techniques, especially phytoremediation. Although some trees such as poplars and willows have been used for phytoremediation, the overall mechanism and roles of all participating organisms are not fully understood [206]. Furthermore, there are still gaps in understanding which plant-derived compounds stimulate the microbial degradation of specific contaminants. For instance, some preliminary results indicate that plant-derived compounds promote the bacterial degradation of *cis*-1,2-dichloroethylene (Fraraccio and Uhlik, unpublished data), which often accumulates as a degradation product of tetrachloroethylene. Such new information could open new avenues for bioremediation research investigating the link between plants and microbial degradative activity.

In recent years, research has expanded beyond the rhizosphere to investigate the role that endophytes—microorganisms living inside plant tissue that do not cause visible harm to the host plant [255,256]—play in bioremediation. Several studies have demonstrated that colonization of plants by endophytes is beneficial due the plant-growth promoting effects of some bacteria. Colonization of plants by such bacteria is important for the growth in a contaminated environment as it leads to the increase of a plant’s resistance or to the decrease in accumulation of the pollutant in the plant due to microbial degradation [257,258,259]. In one case, the endophytes were described to have more degradative genes than were present in the rhizosphere [260], indicating that plants may intentionally attract bacteria harboring degradative genes and provide them with habitat and nutrients. However, very little is still known about SPMEs used for specific communication between plants and their associated bacteria or microbial species selection by plants in the dependence on environmental conditions. These questions could be addressed by SIP experiments designed to track the flow of carbon derived from the plant through the rhizosphere community, looking for patterns of expressed enzymes in both plants and microorganisms, and identifying the role of different metabolites maintaining the relationships.

## Figures and Tables

**Table 1:** An overview of methods used for describing roots and plant–microbe interactions in the rhizosphere.

Object of Study	Method	Reference	Example of Use
root growth and morphology	observation windows + imaging system	[92,93]	[92,93]
	transparent culture media, e.g., Phytagel^TM^ or Nafion^TM^	[94,95]	[95]
	computed tomography (CT)	[97,139]	[97,139]
	magnetic resonance (MRI)	[99,140]	[99,140]
	neutron radiography	[101]	[101]
nutrient transport	magnetic resonance (MRI)	[103]	[103]
	neutron tomography	[102]	[102]
	^11^C-positron emission tomography (^11^C-PET)	[104]	[141]
	optode	[106,107,108]	[105,106,107,108]
	radioisotope labelling (RIP)	[22]	[22]
	stable isotope labelling (SIP)	[127]	[142,143]
interactions plant–microbes	biosensors	[110]	[111,112,113,114,115,116,117,118,119,120,121]
	fluorescence in situ hybridization	[122]	[122,124,125,126]
	metagenomics	[134]	[63,144,145,146]
	metatranscriptomics	[135]	[147,148,149,150]
	metaproteomics	[151]	[152,153,154]
	metabolomics	[155]	[155,156]

**Table 2:** Examples of treatments that led to changes in microbial community structure and activity towards contaminants.

Contaminant	Treatment	Observed Effect	Reference
**PCBs**	limonene	reduction in diversity of bacterial community;	[63]
		community dominated by *Hydrogenophaga*;	
		*Azoarcus* and *Hydrogenophaga* dominated utilization of 4-chloro-^13^C-biphenyl;	
	naringin	reduction of diversity of bacterial community;	
		*Hydrogenophaga* dominated utilization of 4-chloro-^13^C-biphenyl;	
	caffeic acid	largest reduction in diversity of bacterial community;	
		*Burkholderia* dominated utilization of 4-chloro-^13^C-biphenyl;	
		degradation of higher-chlorinated PCBs	
**PCBs**	orange peel	complete mineralization of PCBs;	[61]
		increased abundance of cultivable biphenyl-utilizing bacteria;	
	ivy leaves	complete mineralization of PCBs;	
		increased abundance of cultivable biphenyl-utilizing bacteria;	
	eucalyptus leaves	complete mineralization of PCBs;	
		increased abundance of cultivable biphenyl-utilizing bacteria	
**PCBs**	grapefruit peel	reduction in diversity of bacterial community;	this paper
		*Hydrogenophaga*, *Caulobacter*, and *Skermanella* dominated utilization of 4-chloro-^13^C-biphenyl;	
		*Azotobacter* dominated utilization of 4-chloro-^13^C-biphenyl;	
		increased abundance of cultivable biphenyl-utilizing bacteria;	
	lemon peel	largest reduction in diversity of bacterial community;	
		*Nocardioides* dominated utilization of 4-chloro-^13^C-biphenyl;	
		*Skermanella* dominated utilization of 4-chloro-^13^C-biphenyl;	
		increased abundance of cultivable biphenyl-utilizing bacteria;	
	pears	reduction in diversity of bacterial community;	
		*Azotobacter* dominated utilization of 4-chloro-^13^C-biphenyl;	
		increased abundance of cultivable biphenyl-utilizing bacteria	
**PCBs**	horseradish	*Hydrogenophaga* dominated utilization of ^13^C-biphenyl	[202]
**PCBs**	Austrian pine	increased abundance of cultivable biphenyl-utilizing bacteria	[203]
	ash		
	weeping birch		
	goat willow		
	black locust		
**PCBs**	horseradish	microbial populations of the root zone of each plant significantly differed from one another and/or from the bulk soil	[204]
	black nightshade		
	tobacco		
**diesel and crude oil**	annual ryegrass	enhanced bioremediation	[205]
	red fescue		
**diesel oil**	Alaskan willow	willow had a significant role in structuring the total bacterial community and resulted in significant decreases in diesel range organics	[206]
**PCBs**	nitrogen-rich	fungus *Pleurotus ostreatus* disposes of PCB-degradation activity	[189]
	nitrogen-limiting		
**PCBs**	nitrogen-limiting	fungus *Phanerochaete chrysosporium* disposes of PCB-degradation activity	[190]
**PCBs**	naringin	bacterium *Cupriavidus necator* H850 disposes of PCB-degrading activity while grown on the compounds as carbon sources	[16]
	apigenin		
	catechin		
	morin		
	salicylic acid		[167]
**PCBs**	myricetin	bacterium *Burkholderia xenovorans* LB400 disposes of PCB-degrading activity	[16]
	catechin		
	chrysin		
**PCBs**	limonene	bacterium *Pseudomonas stutzeri* disposes of PCB-degrading activity while grown on the compounds as carbon sources	[168]
	carvone		
**PCBs**	*Mentha spicata* (carvone)	bacterium *Arthrobacter* sp. B1B disposes of PCB-degrading activity while grown on the compound as a carbon source	[207,208]
**4-chlorobiphenyl (PCB 3)**	*Arabidopsis thaliana* exudates (flavanone)	bacterium *Rhodococcus erythropolis* U23A disposes of PCB-degrading activity while grown on the exudates as a carbon source	[85]
**PAHs**	radish (terpenes, salicylic acid)	enhanced bioremediation	[209]
	potato		
	carrot		
	celery		
**PAHs**	not specified	fungus *Phanerochaete chrysosporium* disposes of PAH-degrading activity	[194]
**PAHs**	not specified	fungus *Irpex lacteus* disposes of PAH-degrading activity	[192,193]
**PAHs**	nitrogen-rich	fungus *Lentinus tigrinus* disposes of PAH-degradation activity	[197]
**PCP**	not specified	fungus *Pleurotus ostreatus* disposes of PCP-degradation activity	[201]
**PCP**	not specified	fungus *Irpex lacteus* disposes of PCP-degradation activity	[201]
**PCP**	not specified	fungus *Trametes versicolor* disposes of PCP-degradation activity	[201]
**PCP**	not specified	fungus *Bjerkandera adusta* disposes of PCP-degradation activity	[201]
**PCP**	carvone	bacterium *Arthrobacter* sp. B1B disposes of PCP-degradation activity	[210]
**4-chlorophenol (4-CP)**	leaves of *Phaseolus vulgaris*	bacterium *Arthrobacter chlorophenolicus* A6 disposes of 4-CP-degradation activity	[211]
**TCE**	cumene	bacterium *Rhodococcus gordoniae* P3 disposes of TCE-degradation activity	[212]
**TCE**	cumene	bacterium *Pseudomonas* sp. JR1 disposes of TCE-degradation activity	[213]
**TCE**	cumene	bacterium *Rhodococcus erythropolis* BD1 disposes of TCE-degradation activity	[213]
**TNT**	not specified	fungus *Pleurotus ostreatus* disposes of TNT-degradation activity	[199]
**TNT**	not specified	fungus *Phanerochaete sordida* disposes of TNT-degradation activity	[199]
**TNT**	not specified	fungus *Phlebia brevispora* disposes of TNT-degradation activity	[199]
**TNT**	not specified	fungus *Cyathus stercoreus* disposes of TNT-degradation activity	[199]
**lindane**	intermediate nitrogen concentration	fungus *Pleurotus ostreatus* disposes of lindane-degradation activity	[200]
**dioxins**	not specified	fungus *Panellus stipticus* 99–334 disposes of dibenzo-*p*-dioxins-degradation activity	[198]

PCBs, polychlorinated biphenyls; PAHs, polyaromatic hydrocarbons; PCP, pentachlorophenol; TCE, trichloroethylene; TNT, trinitrotoluene.

## Abbreviations

SPMEs	secondary plant metabolites
PAHs	polycyclic aromatic hydrocarbons
PCBs	polychlorinated biphenyls
L*M*_W_	low molecular weight
L*M*_W_-C	low molecular weight carbon containing
H*M*_W_	high molecular weight
LiP	lignin peroxidase
MnP	manganese peroxidase
CT	computed tomography
MRI	magnetic resonance imaging
PET	positron emission tomography
FISH	fluorescence in situ hybridization
FISH-MAR	fluorescence in situ hybridization combined with microradiography
RIP	radioisotope probing
PLFA	phospholipid-derived fatty acids
SIP	stable isotope probing
VP	versatile peroxidase
DCP	dichlorophenol
CP	chlorophenol
TNT	trinitrotoluene

## Appendix A

### A.1. Description of Our Experiment

The long-term PCB contaminated soil was collected in the dumpsite in Lhenice in 2009. The soil was mainly contaminated by the mixtures of PCBs Delor 103 and 106 with level of chlorination similar to the Aroclor 1242 and 1260, respectively [261]. The amount of natural materials used was calculated in order to achieve the concentration of limonene, naringin or caffeic acid corresponding to 2 g per g of the soil, as was used in our previous study [63]. In order to avoid any effect caused by food additives, the natural products were washed with detergent prior to homogenization. The homogenized plant materials were added to 1.5 kg of soil per sample repeatedly every six weeks for 18 weeks. After the end of the incubation time, bacterial diversity was assessed using pyrosequencing of 16S rRNA gene amplicons in the same way as described elsewhere [262] and using cultivation on biphenyl as a sole carbon source [263]. From samples collected at the same time point, microcosms for stable isotope probing with ^13^C-4-chloro-biphenyl and ^13^C-benzoate were constructed and incubated for 14 days and microbial diversity was assessed in the same way as total communities [262].
